# Armoring chimeric antigen receptor (CAR) T cells as micropharmacies for cancer therapy

**DOI:** 10.1016/j.iotech.2024.100739

**Published:** 2024-09-25

**Authors:** C. Carcopino, E. Erdogan, M. Henrich, S. Kobold

**Affiliations:** 1 Division of Clinical Pharmacology, Department of Medicine IV, University Hospital, Ludwig Maximilian University (LMU) of Munich, Munich, Germany; 2 German Cancer Consortium (DKTK), Partner Site Munich, a partnership between the DKFZ Heidelberg and the University Hospital of the LMU, Heidelberg, Germany; 3 Einheit für Klinische Pharmakologie (EKLiP), Helmholtz Zentrum München - German Research Center for Environmental Health, Neuherberg, Germany

**Keywords:** micropharmacies, CAR-T-cell therapy, BiTEs, TRUCKs, next-generation CARs, immuno-oncology

## Abstract

Chimeric antigen receptor (CAR)-T-cell therapy has emerged as a powerful weapon in the fight against cancer. However, its efficacy is often hindered by challenges such as limited tumor penetration, antigen escape, and immune suppression within the tumor microenvironment. This review explores the potential of armored CAR-T cells, or ‘micropharmacies’, in overcoming these obstacles and enhancing the therapeutic outcomes of adoptive T-cell (ATC) therapy. We delve into the engineering strategies behind these advanced therapies and the mechanisms through which they improve CAR-T-cell efficacy. Additionally, we discuss the latest advancements and research findings in the field, providing a comprehensive understanding of the role of armored CAR-T cells in cancer treatment. Ultimately, this review highlights the promising future of integrating micropharmacies into ATC therapy, paving the way for more effective and targeted cancer treatments.

## Highlights

• Armored CAR-T cells bypass the limitations of immunotherapy.
• Local cytokine secretion enhances T-cell proliferation and persistence.
• Digestive enzymes can be used to increase tumor penetration and infiltration.
• Antibody-like fragment-secreting T cells enhance target recognition and killing.

## Introduction

Chimeric antigen receptor (CAR) T-cell therapy has emerged as a promising avenue in cancer treatment, offering potential solutions for patients facing refractory malignancies. This innovative immunotherapy involves modifying patients’ own T cells to express synthetic receptors, enabling them to selectively target and eliminate cancer cells.^1^ Particularly noteworthy are its successes in hematological malignancies such as B-cell acute lymphoblastic leukemia (B-ALL) and diffuse large B-cell lymphoma (DLBCL).^2^^,^^3^ A total of six CAR-T-cell products are now approved exclusively in B-cell-derived malignancies.^4^ Importantly beyond anecdotal evidence, CAR-T cells have yet to demonstrate their clinical potential in patients suffering from solid tumors.^5^

**Figure 1:**
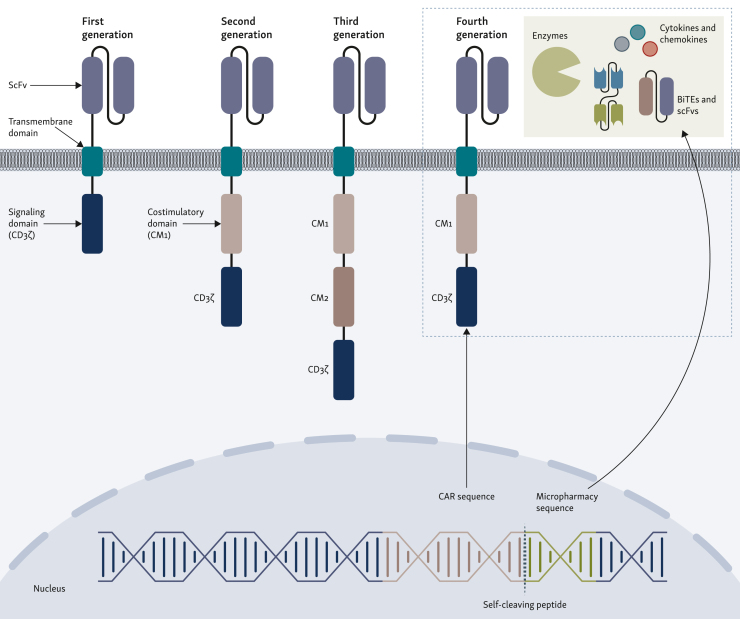
**Structural evolution of chimeric antigen receptors (CARs) across four generations.** First-generation CAR-T cells comprise an extracellular single-chain variable fragment (ScFv) for antigen recognition, a transmembrane domain, and an intracellular signaling domain (CD3ζ). Lacking costimulatory domains, this generation has limited efficacy and persistence. Second-generation CAR-T cells incorporate one costimulatory domain (CM1), such as CD28 or 4-1BB, along with the CD3ζ signaling domain, enhancing T-cell activation and persistence. Third-generation CAR-T cells feature two costimulatory domains (CM1 and CM2), further improving T-cell functionality and longevity. Fourth-generation CAR-T cells, known as TRUCKs (T cells redirected for universal cytokine killing), include additional elements such as cytokines or other molecules (micropharmacies) linked to the CAR sequence via a self-cleaving peptide to enhance therapeutic responses, particularly within the tumor microenvironment. BiTEs, bispecific T-cell engagers.

CARs are engineered proteins that enable T cells to recognize and attack specific targets. The building blocks of CARs include an extracellular antigen recognition domain derived from a monoclonal antibody, a hinge region, a transmembrane domain, and one or more intracellular signaling domains. The antigen recognition domain typically consists of a single-chain variable fragment (scFv) binding to a specific antigen. The hinge and transmembrane regions anchor the CAR to the T-cell membrane, while the intracellular signaling domains activate the T cell upon antigen binding (Figure 1).^6^

CAR-T cells have evolved through different generations to improve their efficacy and persistence. First generations contain a single intracellular signaling domain, usually CD3ζ, initiating T-cell activation. The second generation includes an additional costimulatory domain, such as CD28 or 4-1BB, which enhances T-cell proliferation and survival. Third-generation CAR-T cells incorporate two costimulatory domains, further boosting their antitumor activity. The latest fourth-generation CAR-T cells are engineered to deliver additional immune-modulating agents to the tumor microenvironment (TME), enhancing their ability to eradicate cancer cells and overcome resistance mechanisms.^7^ Numerous new CAR designs have been proposed in recent years. However, due to the lack of systematic comparative studies investigating these constructs against each other, it is uncertain whether any of these new designs offer significant improvements over existing ones. These have been reviewed extensively by us and others. We invite the interested reader to refer to these overviews.^8^, ^9^, ^10^

Despite its promise, CAR-T-cell therapy faces several challenges and limitations that warrant careful consideration. One prominent challenge is the complexity of manufacturing CAR-T cells. The process involves collecting patients’ T cells through leukapheresis, genetically modifying them *ex vivo* to express CARs, expanding the modified cells to therapeutic doses, and finally reinfusing them back into patients. Each step of this manufacturing process is intricate and resource-intensive, posing logistical hurdles and contributing to high therapy cost.^11^

Another relevant limitation is the occurrence of adverse events, including cytokine release syndrome (CRS) and neurotoxicity. CRS, characterized by systemic inflammation, arises from massive release of cytokines following CAR-T-cell activation. Although CRS can often be managed with supportive care, severe cases may necessitate intensive interventions, underscoring the importance of developing strategies to mitigate its severity and incidence.^12^ Neurotoxicity, on the other hand, manifests as cognitive disturbances, seizures, and cerebral edema, posing additional challenges to patient management and necessitating close monitoring and prompt intervention.^13^^,^^14^

**Figure 2:**
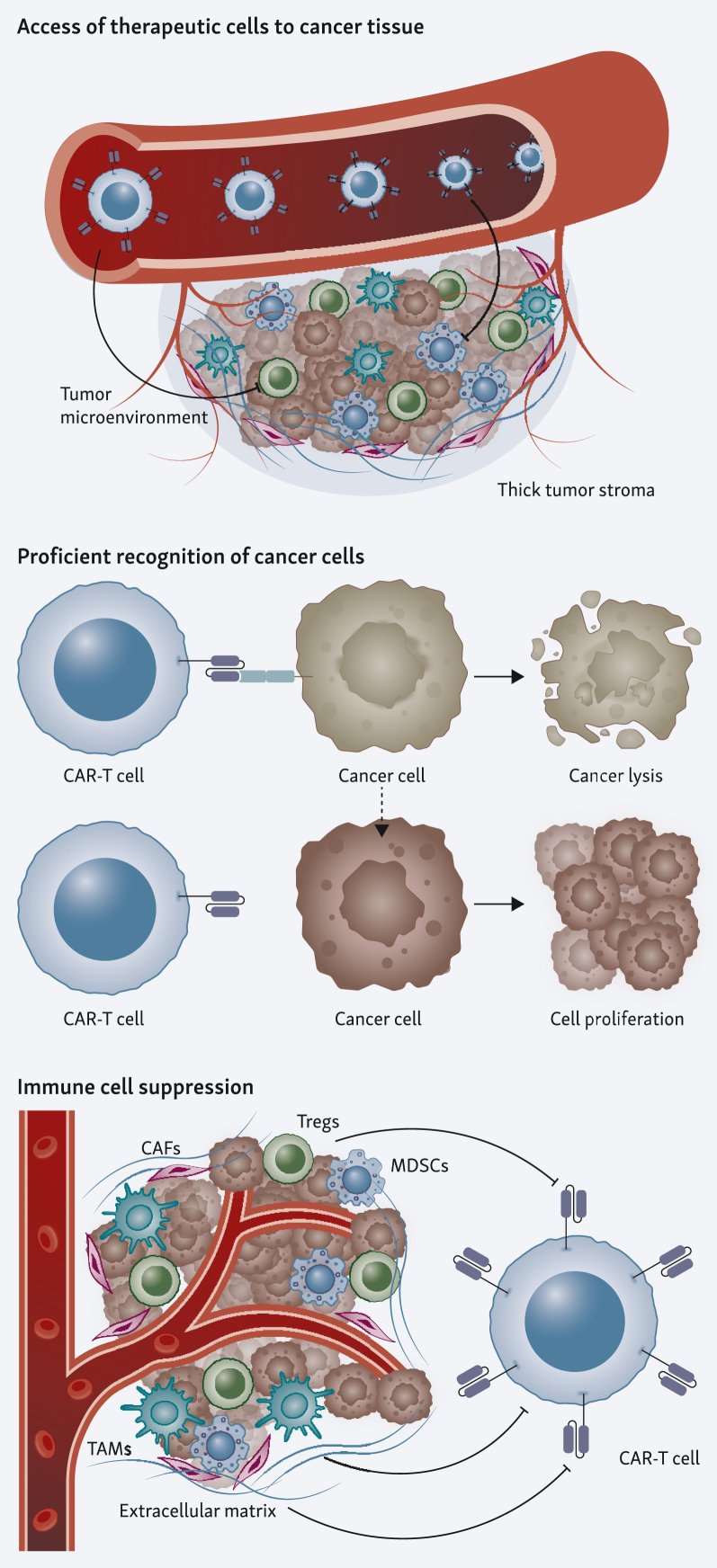
**Challenges of conventional CAR-T-cell therapy.** Access of therapeutic cells to cancer tissue: The top-left panel illustrates the difficulty CAR-T cells face in infiltrating the tumor Proficient recognition of cancer cells: The top-right panel demonstrates the failure to recognize cancer cells accurately that can result in cancer cell proliferation. Immune cell suppression: The bottom panel highlights the immunosuppressive elements within the TME that inhibit CAR-T-cell activity. Various suppressive cells such as TAMs, MDSCs, Tregs, and CAFs contribute to the immunosuppressive milieu. CAFs, cancer-associated fibroblasts; CAR, chimeric antigen receptor; ECM, extracellular matrix; MDSCs, myeloid-derived suppressor cells; TAMs, tumor-associated macrophages; TME, tumor microenvironment; Tregs, regulatory T cells.

Efficacy of CAR-T-cell therapy, particularly in solid tumors, is hindered by three key factors as described in Figure 2: firstly, the access of therapeutic cells to cancer tissue; secondly, proficient recognition of cancer cells; and lastly, immune cell suppression. The immunosuppressive TME and physical barriers, including tumor stroma, obstruct penetration and movement of CAR-T cells. A strategy to overcome tumor infiltration within solid tumors is local administration of CAR-T cells including intrapleural and intrathecal delivery.^15^^,^^16^ However, although some studies have shown improved efficacy and reduced on-target-off-tumor toxicity,^17^^,^^18^ the approach is currently limited to single tumor lesions.^19^ An alternative strategy is engineering of CAR with chemokine receptors for targeted tumor homing.^20^, ^21^, ^22^, ^23^ Another challenge arises from antigen escape, which refers to partial or complete loss of target antigen expression due to genetic diversity or evolving resistance. This has been observed also in solid tumors in addition to ALL.^24^, ^25^, ^26^ To reduce disease relapse via antigen escape mechanisms, numerous strategies are developed based on simultaneous targeting of multiple antigens,^27^, ^28^, ^29^, ^30^ but still with potential for enhancement. Tumor-promoting cytokines, chemokines, and growth factors are being synthesized by many cell types, including myeloid-derived suppressor cells, tumor-associated macrophages, regulatory T cells (Tregs), and tumor cells, resulting in an immunosuppressive TME.^31^ Additionally, immune checkpoint pathways such as programmed cell death protein 1 (PD-1) or cytotoxic T-lymphocyte associated protein 4 can act to suppress antitumor immune responses. One major origin of CAR-T-cell therapy failure is poor T-cell expansion and short-term persistence. A hypothesis being that T-cell exhaustion may be initiated by co-inhibitory pathways.^32^ To counteract this, a combined immunotherapy of CAR-T cells and checkpoint blockade is thought to be the next immunotherapy frontier, where CAR-T cells providing cytotoxicity and PD-1/ programmed death-ligand 1 (PD-L1) blockade ensuring effector T-cell functions.^33^ But the redundancy of immune checkpoints may call for additional approaches to make up for compensation. Immune evasion through distinct biological pathways underscores the need for novel approaches, which have been reviewed extensively in recent years.^22^^,^^34^

To circumvent the aforementioned limitations introduced by conventional therapeutic T-cell products, new generations of CAR-T cells with improved competence are being developed.^35^^,^^36^ One distinct example is ‘armored CAR-T cells’ where the T cells are engineered in a way not only to express a CAR but also to secrete localized doses of therapeutic cytokines, immunostimulatory ligands, antibody-like proteins, or small-molecules into the TME.^37^, ^38^, ^39^ These cell-mediated drug delivery methods, or ‘cellular micropharmacies’, represent a revolutionary approach to key hurdles and also render drug administration more controlled and selective.^40^ Engineering armored CAR-T cells resembles the production of previous generations in many aspects. For a lion’s share of micropharmacies, the cDNA of the additional ‘weapon’ is first subcloned into the CAR cassette either in an inducible or constitutive expression manner (see Table 1), which can be linked via a self-cleaving peptide,^41^, ^42^, ^43^, ^44^, ^45^ or an internal ribosome entry site,^46^ enabling translation of two separate proteins. To date, the self-cleaving peptides P2A and T2A are the most frequently used for this purpose.

**Table 1:** Comparison of the advantages and disadvantages of constitutive and inducible expression systems^47^, ^48^, ^49^

	Constitutive	Inducible
Advantage	• Continuous therapeutic effect • Simplified control mechanism • Persistent immune stimulation • Potentially stronger initial response	• Targeted and controlled therapeutic release • Reduced potential for toxicity and higher safety • Less T-cell exhaustion • More dynamic therapy
Disadvantage	• Associated with overstimulation and cytotoxicity • Higher T-cell exhaustion • Lack of therapeutic precision and control in case of undesired effects	• Complex vector design and additional genetic components • Potential delays in response time • May cause inconsistent expression levels

**Figure 3:**
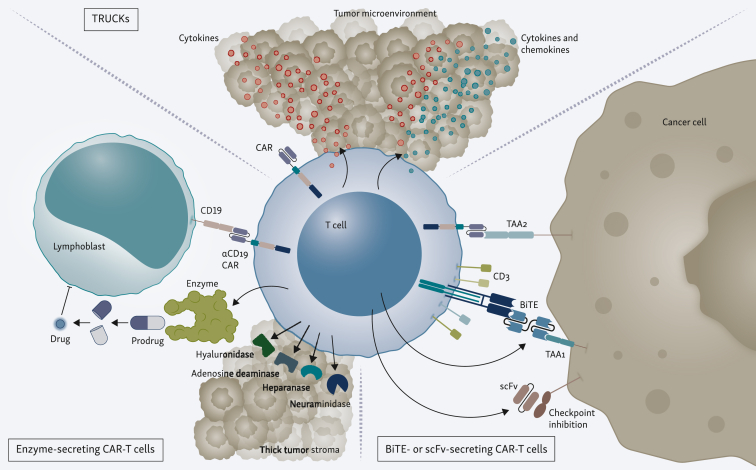
**Advanced strategies in CAR-T-cell therapy: micropharmacies.** TRUCKs (T cells redirected for universal cytokine killing): The top section shows CAR-T cells engineered to secrete cytokines and chemokines upon antigen recognition, enhancing the immune response within the TME. Enzyme-secreting CAR-T cells: The bottom-left section depicts CAR-T cells engineered to secrete enzymes such as hyaluronidase, adenosine deaminase, heparanase, and neuraminidase. These enzymes degrade components of the thick tumor stroma, facilitating CAR-T-cell penetration and access to cancer cells. BiTE- or scFv-secreting CAR-T cells: The bottom-right section illustrates CAR-T cells designed to secrete BiTEs or scFvs. These secreted molecules can redirect T cells to cancer cells, enhancing target cell recognition and killing. Additionally, these CAR-T cells can be engineered to inhibit immune checkpoints, thereby preventing immune suppression and boosting T-cell activity. BiTEs, bispecific T-cell engagers; CAR, chimeric antigen receptor; ScFvs, single-chain variable fragments; TAA, tumor-associated antigen.

Here, we examine the term ‘targeted cellular micropharmacies’ to encompass a wide range of cellular therapies, engineered to selectively deliver therapeutic payloads to diseased tissue environments (Figure 3). This approach aims to enhance efficacy of conventional cellular therapies, improve therapeutic indices of drugs, or provide completely new pharmacological impacts through controlled delivery of therapeutic agents.^50^ Therefore, the main scope of this review is to gather insights into the latest developments, particularly focusing on micropharmacies, within CAR-T-cell therapy to address the obstacles and propel the field forward.

### Cytokine modulation

A major strategy deployed to circumvent an immunosuppressive TME and thereby increasing CAR-T-cell efficacy is T cells redirected for universal cytokine-mediated killing (TRUCKs). These are fourth-generation CAR-T cells engineered to secrete stimulatory cytokines to target tumor tissue.^51^ Currently, cytokines interleukin (IL)-7, IL-12, IL-15, IL-18, IL-22, and IL-23 are being investigated in great detail and have already been compared in different reviews,^37^^,^^52^ hence we will focus on the latest studies and discoveries. In general, TRUCKs must be delimited from CAR-T cells that are expressing an additional membrane-bound cytokine, such as IL-12^53^ or IL-15.^54^

Among these cytokines, serving as linkers between innate and adaptive immunity, IL-12 stands out as one of the first to be utilized in TRUCK settings. IL-12 modulates the TME by inducing other cytokines including interferon (IFN)-γ and tumor necrosis factor (TNF)-ɑ, improving Th1-type response and suppressing tumor-induced Treg cell proliferation.^55^ Already in 2014, this approach was used to engineer Mucin-16 (Muc16) targeting CAR-T cells to additionally secrete IL-12, leading to enhanced CAR-T-cell proliferation and prolonged persistence in an IFN-γ-dependent manner in ovarian cancer *in vivo* models.^46^ Similar studies proved higher survival in syngeneic mouse models.^56^ In the following years, IL-12 was inserted into anti-glypican-3 (GPC3) CAR-T cells, this time in an inducible system by setting the cytokine under the control of the nuclear factor of activated T cells promoter. By applying those TRUCKs to hepatocellular carcinoma (HCC) xenograft as well as syngeneic tumor models, a stronger killing efficacy was detected compared to CAR-T cells alone.^57^ Additionally, this inducible system did not show any toxicities in murine tumor models.^57^^,^^58^ In contrast, first clinical studies showed that high serum IL-12 levels correlated with clinical toxicities.^59^ Therefore Zhou et al. developed armed anti-CD19 CARs expressing hypoxia-regulated IL-12, meaning that IL-12 is only synthesized under hypoxic conditions, and were able to show *in vivo* that this design boosted the CAR function as previously explained, but with fewer side-effects.^60^

The second frequently studied cytokine is IL-18, which also induces expression of IFN-γ and TNF-ɑ. IL-18 acts as an activator of monocytes and lymphocytes, and a recruiter of different immune cell subsets.^61^ First studies from 2017, carried out in syngeneic and xenograft acute myeloid leukemia (AML) models, have already demonstrated that CAR-T cells secreting constitutive IL-18 boost their proliferation and antitumor activity compared to anti-CD19 or anti-mesothelin CARs alone by recruiting a second wave of immune cells to the TME.^41^ Very recently, Jaspers et al. designed TRUCKs combining delta-like ligand 3 (DLL3) antigen with the mature form of IL-18, again leading to permanent IL-18 secretion. This setup demonstrated that IL-18 does not only enhance activation of engineered but also endogenous T cells, thereby inducing a memory phenotype of those cells, both in murine and human models. A direct comparison to IL-12, whereby the manufacturing process was similar to anti-DLL3-IL-18 CAR-T cells, was able to point out a more robust antitumor response of IL-18 due to higher proliferation and persistence of CAR-T cells.^62^ Lately it has also been shown that IL-18 TRUCKs can be produced in a good manufacturing practice-compliant process.^63^

By regulating activation and proliferation of T cells, among others, the lymphocyte growth cytokine IL-15 became a further important candidate^64^ for equipping CAR-T cells. Firstly, combinatorial studies were already conducted in 2010 in B-cell lymphoma by inserting IL-15 in anti-CD19 CAR-T cells, leading to greater expansion, survival, and enhanced antitumor activity *in vivo*. To circumvent IL-15-induced toxicities *in vivo*, an inducible caspase-9 gene (iC9) safety switch was included.^65^ A few years later, Chen et al. equipped GD2-CAR-T cells with the iC9-IL-15 construct and could confirm previous findings. Armed CAR-T cells had a less exhausted but rather stem cell-like phenotype, and importantly, this high *in vivo* antitumor activity did not correlate with any toxicities known to be critical for systemic IL-15 administration.^66^ An alternative way to circumvent possible *in vivo* toxicities of too high IL-15 concentrations was conducted by not only inserting IL-15 into the CAR constructs but also its receptor IL-15Rα.^67^ Also, recent studies in gastric and pancreatic cancer models could verify that CAR-T cells armored with IL-15 experience greater T-cell expansion and faster tumor infiltration by limiting angiogenesis.^68^ Nonetheless, all those studies focused on short-term effects, whereas long-term combinatorial effects still need to be elucidated.^42^ Moreover, it remains questionable how predictive mouse models are to assess toxicities. For more certainty, the safety of those TRUCKS must be carefully assessed in a phase I dose-escalation design.^69^

Another recent approach introduced the STAT3-activating cytokine IL-23 into CAR-T cells. Its role in tumor cell growth is still controversial, as low amounts may promote inflammation and early tumor initiation,^70^ whereas high levels cause antitumor effects by recruiting memory T and Th17 cells.^71^ IL-23 is a two-subunit (p19 and p40) cytokine,^72^ whereby only p19 gets activated upon T-cell receptor (TCR) stimulation. By engineering CAR-T cells to exclusively express the p40 subunit, cytokine spread is restricted, ensuring that IL-23 is assembled solely upon T-cell activation within the TME. Ma et al. used this approach and demonstrated an increased antitumor activity as well as sustained T-cell persistence in different neuroblastoma and pancreatic tumor models. Autocrine IL-23 production of this system specifically enhanced local effects and had no discernible impact on the immune system, which, according to the authors, increases safety compared to IL-15 or IL-18 TRUCKs.^69^

### Next generation of TRUCKs

Latest approaches no longer concentrate exclusively on equipping CAR-T cells with cytokines, but generate TRUCKs expressing chemokines, by adding P2A to the CAR cassette.^73^, ^74^, ^75^ Chemokines are small G-protein-coupled proteins that are involved in the regulation of cell migration.^76^ Due to their ability to increase accumulation of antitumor effector cells within the tumor, various chemokine receptors have already been combined with CAR-T cells alone.^77^ First attempts to co-arm CAR-T cells were conducted in 2020 by Luo et al. equipping anti-CLDN18.2 CAR-T cells with both IL-7 and CCL21. In three different solid tumor models, they were able to show better proliferation and chemotaxis, as well as higher infiltration of CARs and dendritic cells.^73^ Recently, IL-15 TRUCKs were equipped with chemokines. For instance, 19 x 15 CARs (TRUCKs additionally equipped with CCL19) showed a higher cytotoxicity towards investigated gastric cancer (GC) lines in a zebrafish model, reduced expression of T-cell exhaustion markers, as well as better chemotaxis.^74^ Consistent studies with 10 × 15 CARs, armored with IL-15 and CXCL10, displayed synergistic effects in GC murine *in vivo* models through long-lasting cytotoxic effects and promoted long-term T-cell recruitment.^75^ Although none of the aforementioned studies directly compared co-armed TRUCKs with regular TRUCKs, the individual effects appear to be reciprocally beneficial in the treatment of preclinical solid tumor models.

### BiTE-secreting CAR-T cells

Bispecific T-cell engagers (BiTEs) or T-cell-engaging antibody molecules (TEAMs) are structured with two arms composed of scFVs. One scFV is designed to engage T cells through CD3 binding, leading to T-cell activation. The other scFV is designed to bind a specific antigen present on tumor cells, so called tumor-associated antigens. This dual-arm structure allows BiTE to connect T cells to tumor cells, facilitating a targeted immune response against the tumor, while reducing non-specific activity.^78^

In 2015, Iwahori et al. engineered the first T cells secreting BiTEs that were designed to enable a targeted immune response against the ephrin type-A receptor 2 (EphA2), which is expressed on glioblastoma, breast, prostate, and lung tumor cells. The main advantage of this approach resides in redirecting non-CAR-engineered naturally occurring T cells present in the TME (bystander T cells) towards tumor cells. This recruitment greatly amplifies the overall immune response against the tumor, leading to potent antitumor activity in xenograft models. However, the concept faces limitations, notably the BiTEs’ short half-life, which does not allow for self-amplification but may be advantageous in limiting BiTE impact outside of CAR and tumor vicinity.^43^

Building on these promising findings, Choi et al. extended the scope in 2019 by targeting both wild-type epidermal growth factor receptor (EGFR) and mutated EGFRvIII antigens. Their goal was to enhance targeting of heterogeneous tumors like glioblastoma and mitigate antigen loss in EGFRvIII-negative contexts. Notably, Choi et al. demonstrated that Tregs could also be redirected to function as cytotoxic killer cells.^44^ Four years later, they initiated a first-in-human phase I clinical study and recently reported on three patients with glioblastoma. A single intraventricular infusion of dual-targeting CARv3-TEAM-E T cells led to rapid and dramatic tumor regression within days, although this response was transient in two of the three subjects. Notably, no adverse events greater than grade 3 or dose-limiting toxic effect were observed.^79^

Along the same lines, a preclinical study from 2022 leveraged IL13Ra2 to cover a broader spectrum of tumor cells compared to EGFRvIII targeting. RNA-seq analysis revealed increased EGFR and IL13Ra2 expression in high-grade gliomas versus low-grade tumors. BiTE-engineered CAR-T cells outperformed conventional CAR-T cells *in vitro* and early-phase *in vivo* experiments but fell short in demonstrating long-term efficacy. One key aspect may reside in the choice of the model, as the use of NOD SCID gamma (NSG) mice as an *in vivo* model restricted the pool of bystander T cells available for BiTE recruitment. Also, concerns were raised regarding the persistence of BiTE T cells in the long term due to their up-regulation of checkpoint expression after tumor response.^80^

The same year, Cao et al. proposed another innovative strategy by developing anti-GPC3 CAR-T cells secreting BiTEs against B7H3, a B7 family member immune checkpoint protein that is highly expressed in both cancer cells and tumor-infiltrating immune cells. Both GPC3 and B7H3 are strongly expressed antigens in hepatocellular carcinoma. Although the demonstration was limited to *in vitro* experiments, researchers observed that BiTE CAR-T cells effectively recruited un-transduced bystander T cells to GPC3-B7H3+ HCC cells, resulting in potent antitumor activity.^81^

In 2023, CAR-T cells secreting BiTEs were designed to target two tumor antigens highly expressed in ovarian cancer cells: the cell surface Muc16 (4H11) and the intracellular Wilms tumor 1 (ESK1). This strategy demonstrated enhanced anticancer activity against cancer cells with low Muc16 expression, both *in vitro* and *in vivo.*^82^

Huang et al. employed γδT cells engineered with a nanobody-based anti-human leukocyte antigen-G (HLA-G) CAR and a secreted PD-L1/CD3ε BiTE construct (Nb-CAR.BiTE). This innovative approach addresses the challenge of immune escape from anti-HLA-G CAR cell therapy. Notably, γδT effector cells offer natural antitumor activity and allogeneic potential, while nanobodies provide stability, high binding capacity, and genetic manipulation flexibility due to their smaller size.^83^

In a similar approach, Branella et al. have developed anti-c-kit γδ T cells secreting BiTEs for the treatment of AML. This study showed that anti-murine-stem cell factor CAR- and anti-human-SCF BiTE-modified γδ T cells effectively eradicated c-kit+ AML cell lines and sca-1+ murine bone marrow cells *in vitro*. *In vivo*, the survival of NSG mice engrafted with disseminated AML was moderately prolonged by hSCF BiTE-modified γδ T cells. However, therapeutic efficacy was restricted by lack of γδ T-cell homing to murine bone marrow.^84^

Lastly, Wehrli et al. engineered anti-mesothelin CAR-T cells to secrete TEAMs directed against the fibroblast activation protein (FAP). This novel strategy aims to provide a more favorable TME for CAR-T cells to target pancreatic ductal adenocarcinoma (PDAC) by targeting cancer-associated fibroblasts (CAF), which are responsible for PDAC’s thick stroma. These anti-mesoFAP CAR-TEAM cells were shown to effectively eradicate both PDAC and CAF across multiple *in vitro*, *in vivo*, and *ex vivo* patient-derived models.^85^

### scFv-secreting CAR-T cells

The enhancement of CAR-T-cell tumor redirection and recognition of cancer-associated antigens can be achieved by further leveraging the specificity of scFv. Numerous CARs that secrete antibody-like proteins have been developed and already reviewed by others.^37^^,^^86^, ^87^, ^88^ Notably, studies looking into scFv secretion by CARs have mainly focused on checkpoint blockade by secretion of anti-PD-1 scFvs.^89^, ^90^, ^91^ This section will discuss recent advancements in this rapidly evolving field.

In 2022, Wang et al. designed anti-mesothelin CAR-T cells to secrete PD-L1-blocking scFv (termed sec-MesoCAR-T cells) mitigating the immunosuppressive effect of pancreatic cells on CAR-T cells. These sec-MesoCAR-T cells demonstrated enhanced cytotoxicity against the pancreatic cancer model BxPC3, both *in vitro* and *in vivo*, and secreted high levels of IL-2, IL-6, and IFN-γ *in vitro*. They also accumulated extensively at the tumor site *in vivo*, thereby increasing the concentration of PD-L1 antibody.^92^

In 2023, Yang et al. showed that PD-1-blocking scFvs, when specifically delivered to cancer cells via anti-CD133 CAR-T cells, enhanced antitumor efficacy against HCC models both *in vitro* and in xenograft mouse models.^93^

Similarly, Dunn et al. enhanced the efficacy of anti-PD-1 scFv-secreting CAR-T cells by engineering CD28-costimulatory CAR-T cells to secrete a fusion protein containing the soluble trimeric 4-1BB ligand. Both *in vivo* and *in vitro,* anti-CD19 CAR-T cells secreting PD-1 and 4-1BBL scFv showed improved tumor growth inhibition and overall survival. This led to the development of anti-mesothelin CAR-T cells and confirmed the enhanced efficacy against solid tumors of CAR-T cells secreting anti-PD1-4-1BBL.^94^

Another interesting approach involves combining effector T-cell-mediated cell death with vascular targeting of the TME within a single vector system. In a preclinical study, T cells were transduced with an anti-GD2 CAR along with a CAR-inducible transgene encoding for recombinant truncated tissue factor (tTF)-conjugated to the C-terminal peptide GNGRAHA (NGR). Engineered T cells secreting tTF-NGR exerted potent GD2-specific effector functions and secreted tTF-NGR activated the extrinsic coagulation pathway in a strictly GD2-dependent manner. Nevertheless, their antitumor activity was limited in a murine Ewing sarcoma xenograft model.^95^

To modulate CRS, Lin et al. engineered self-regulating anti-CD19 CAR-T cells that secrete scFv derived from tocilizumab, an IL-6 receptor antibody. Tocilizumab-derived scFv secretion disrupted the cytokine feedback loop that typically leads to CRS, reduced toxicity *in vivo* and promoted the enrichment of cytotoxic T cells with memory signatures.^96^

### Enzyme-secreting CAR-T cells

One strategy to overcome the aforementioned hurdles introduced by conventional CAR-T-cell therapy is arming CAR-T cells with enzymes.^87^ Depending on the purpose and type of micropharmacy, CAR-T cells can be engineered to produce different types of enzymes.

Caruana et al. demonstrated that CAR-T cells secreting heparanase (HPSE) can remodel the TME through tissue degradation to infiltrate solid tumors with rich stromal tissue. They combined anti-GD2 (to target neuroblastoma) and anti-chondroitin sulfate proteoglycan-4 CAR-T cells (to target melanoma) with HPSE and showed increased cytotoxicity both *in vitro* and *in vivo*. Additionally, the group demonstrated that enzyme-armed CAR-T cells did not possess preferential accumulation in vital tissues including the lung or liver.^97^

Beyond heparanase, Durgin et al. equipped CAR-T cells with neuraminidase (NA), an enzyme capable of removing sialic acid residues to directly target surface glycans suppressing T-cell functions and demonstrated that NA-secreting CAR-T cells had improved persistence and immunosurveillance across a wide range of tumor models. In addition, the group showed that NA shifted the differentiation of T cells into a naive-like state, resulting in enhanced persistence and prolonged tumor control *in vivo.*^98^

In 2022, Qu et al. engineered CAR-T cells to constitutively express adenosine deaminase 1 (ADA), which catabolizes adenosine into inosine and is crucial for a functional immune system. Their work showed that ADA secretion was neither toxic to CAR-T cells nor did it impede the effector function of the cells *in vitro*. Furthermore, ADA expression lowered percentage of exhausted T cells, increased effector cytokine production such as IFN-γ and TNF-ɑ, and decreased both Treg differentiation and PD-L1 expression. Moreover, ADA permitted superior tumor growth control and increased overall survival as well as higher persistence in tumor, blood, spleen, and bone marrow in their ovarian cancer *in vivo* solid tumor model.^99^

The same year, Zhao et al. constructed CAR-T cells to express hyaluronidase (HAase) for hyaluronic acid degradation, the major component of tumor extracellular matrix. They used this system in combination with anti-CD19 and anti-carcinoembryonic antigen CARs to target A20 and CT26 tumors, respectively, and demonstrated that those CAR-T cells improved infiltration capacity compared to unmodified cells. In these settings, laser confocal microscopy showcased deeper infiltration in HAase-modified compared to unmodified CAR-T cells.^100^

Recently, Gardner et al. developed CAR-T cells that can produce orthogonally acting small-molecule drugs locally at the disease site, the novel strategy termed as synthetic enzyme-armed killer (SEAKER) cells. In this study, 5′-O-sulfamoyl adenosine was picked as the prodrug due to its high toxicity towards cancer cells, and proved that SEAKER cell–prodrug combination exhibited antitumor efficacy at low effector-to-target cell ratios. In an *in vivo* lymphoma model, SEAKER cells exhibited comparable antitumor efficacy to conventional CAR-T cells in the absence of a prodrug, while displaying augmented antitumor effects upon prodrug administration. Subsequent analyses revealed that SEAKER cells expressing markers of exhaustion retained enzymatic activity for the prodrug, indicating extended therapeutic utility of these otherwise functionally impaired cells.^45^

## Conclusion

The development and implementation of armored CAR-T cells represent a significant leap forward in cancer immunotherapy. By integrating additional functionalities into conventional CAR-T cells, the aforementioned limitations can be effectively addressed. These engineered micropharmacies not only enhance the efficacy of CAR-T cells in targeting and eliminating tumor cells but also improve their persistence, penetration, and overall performance within the hostile TME. This performance boost from the addition of the micropharmacy may be due to usage of an inducible system rather than a constitutive one to limit the cytotoxicity.

Despite the promising results obtained from both *in vivo* and *in vitro* studies, the long-term effects and comprehensive clinical trials have only been explored in a limited number of studies, which are displayed in Table 2. Moving forward, strategies demonstrating robust preclinical efficacy, a strong safety profile, and feasible manufacturing processes should be advanced to clinical trials. Moreover, engaging with regulatory bodies early in the development process will be crucial in optimizing these therapies for clinical success. Continued research and carefully designed clinical trials are essential to fully realize the potential of armored CAR-T cells, ensuring their safe and effective application in cancer treatment.

**Table 2:** Clinical trials on micropharmacies in the context of next-generation CAR-T cells

Micropharmacy	Trial ID	Status	Phase
IL12-secreting CAR	NCT06343376	Recruiting	Phase I
	NCT02498912	Study completion 1 August 2025 (Estimated)	
IL18-secreting CAR	NCT06287528	Recruiting	
IL15-secreting CAR	NCT04377932	Primary completion 1 February 2025 Study completion 1 February 2040 (estimated)	
IL15- and IL21-secreting CAR	NCT04715191	Primary completion 1 August 2026 Study completion 3 July 2041	
	NCT06198296	Study start 1 January 2026 (Estimated) Study completion 1 February 2043	
Nanobody-secreting CAR	NCT05373147	Study completion August 2023	
	NCT06249256	Ongoing	
	NCT06248697		
	NCT04503980	Study completion June 2022	
	NCT05089266	Recruiting	
PD-1-secreting CAR	NCT04556669	Recruiting	
	NCT03615313	Unknown	Phase I/II

CAR, chimeric antigen receptor; IL, interleukin; PD-1, programmed cell death protein 1.

## References

[1] June C.H., Sadelain M. (2018). Chimeric antigen receptor therapy. N Engl J Med.

[2] Maude S.L., Laetsch T.W., Buechner J. (2018). Tisagenlecleucel in children and young adults with B-cell lymphoblastic leukemia. N Engl J Med.

[3] Neelapu S.S., Locke F.L., Bartlett N.L. (2017). Axicabtagene ciloleucel CAR T-cell therapy in refractory large B-cell lymphoma. N Engl J Med.

[4] Cappell K.M., Kochenderfer J.N. (2023). Long-term outcomes following CAR T cell therapy: what we know so far. Nat Rev Clin Oncol.

[5] Patel U., Abernathy J., Savani B.N., Oluwole O., Sengsayadeth S., Dholaria B. (2021). CAR T cell therapy in solid tumors: a review of current clinical trials. EJHaem.

[6] Huang R., Li X., He Y. (2020). Recent advances in CAR-T cell engineering. J Hematol Oncol.

[7] Daei Sorkhabi A., Mohamed Khosroshahi L., Sarkesh A. (2023). The current landscape of CAR T-cell therapy for solid tumors: mechanisms, research progress, challenges, and counterstrategies. Front Immunol.

[8] Maalej K.M., Merhi M., Inchakalody V.P. (2023). CAR-cell therapy in the era of solid tumor treatment: current challenges and emerging therapeutic advances. Mol Cancer.

[9] Mitra A., Barua A., Huang L., Ganguly S., Feng Q., He B. (2023). From bench to bedside: the history and progress of CAR T cell therapy. Front Immunol.

[10] Dagar G., Gupta A., Masoodi T. (2023). Harnessing the potential of CAR-T cell therapy: progress, challenges, and future directions in hematological and solid tumor treatments. J Transl Med.

[11] Levine B.L., Miskin J., Wonnacott K., Keir C. (2016). Global manufacturing of CAR T cell therapy. Mol Ther Methods Clin Dev.

[12] Frey N., Porter D. (2019). Cytokine release syndrome with chimeric antigen receptor T cell therapy. Biol Blood Marrow Transplant.

[13] Wang Z., Han W. (2018). Biomarkers of cytokine release syndrome and neurotoxicity related to CAR-T cell therapy. Biomark Res.

[14] Hay K.A., Hanafi L.A., Li D. (2017). Kinetics and biomarkers of severe cytokine release syndrome after CD19 chimeric antigen receptor–modified T-cell therapy. Blood.

[15] Adusumilli P.S., Zauderer M.G., Rivière I. (2021). A phase I trial of regional mesothelin-targeted CAR T-cell therapy in patients with malignant pleural disease, in combination with the anti-PD-1 agent pembrolizumab. Cancer Discov.

[16] Gargett T., Ebert L.M., Truong N.T.H. (2022). GD2-targeting CAR-T cells enhanced by transgenic IL-15 expression are an effective and clinically feasible therapy for glioblastoma. J Immunother Cancer.

[17] Priceman S.J., Tilakawardane D., Jeang B. (2018). Regional delivery of chimeric antigen receptor-engineered T cells effectively targets HER2^+^ breast cancer metastasis to the brain. Clin Cancer Res.

[18] Brown C.E., Aguilar B., Starr R. (2018). Optimization of IL13Rα2-targeted chimeric antigen receptor T cells for improved anti-tumor efficacy against glioblastoma. Mol Ther.

[19] Sterner R.C., Sterner R.M. (2021). CAR-T cell therapy: current limitations and potential strategies. Blood Cancer J.

[20] Rapp M., Grassmann S., Chaloupka M. (2015). C-C chemokine receptor type-4 transduction of T cells enhances interaction with dendritic cells, tumor infiltration and therapeutic efficacy of adoptive T cell transfer. Oncoimmunology.

[21] Cadilha B.L., Benmebarek M.R., Dorman K. (2021). Combined tumor-directed recruitment and protection from immune suppression enable CAR T cell efficacy in solid tumors. Sci Adv.

[22] Lesch S., Benmebarek M.R., Cadilha B.L. (2020). Determinants of response and resistance to CAR T cell therapy. Semin Cancer Biol.

[23] Grover N.S., Ivanova A., Moore D.T. (2021). CD30-directed CAR-T cells co-expressing CCR4 in relapsed/refractory Hodgkin lymphoma and CD30+ cutaneous T cell lymphoma. Blood.

[24] Majzner R.G., Mackall C.L. (2018). Tumor antigen escape from CAR T-cell therapy. Cancer Discov.

[25] Maude S.L., Teachey D.T., Porter D.L., Grupp S.A. (2015). CD19-targeted chimeric antigen receptor T-cell therapy for acute lymphoblastic leukemia. Blood.

[26] Brown C.E., Alizadeh D., Starr R. (2016). Regression of glioblastoma after chimeric antigen receptor T-cell therapy. N Engl J Med.

[27] Hege K.M., Bergsland E.K., Fisher G.A. (2017). Safety, tumor trafficking and immunogenicity of chimeric antigen receptor (CAR)-T cells specific for TAG-72 in colorectal cancer. J Immunother Cancer.

[28] Lin Q., Zhao J., Song Y., Liu D. (2019). Recent updates on CAR T clinical trials for multiple myeloma. Mol Cancer.

[29] Wilkie S., van Schalkwyk M.C.I., Hobbs S. (2012). Dual Targeting of ErbB2 and MUC1 in breast cancer using chimeric antigen receptors engineered to provide complementary signaling. J Clin Immunol.

[30] Hegde M., Mukherjee M., Grada Z. (2016). Tandem CAR T cells targeting HER2 and IL13Rα2 mitigate tumor antigen escape. J Clin Invest.

[31] Quail D.F., Joyce J.A. (2013). Microenvironmental regulation of tumor progression and metastasis. Nat Med.

[32] Yin Y., Boesteanu A.C., Binder Z.A. (2018). Checkpoint blockade reverses anergy in IL-13Rα2 Humanized scFv-Based CAR T cells to treat murine and canine gliomas. Mol Ther Oncol.

[33] Grosser R., Cherkassky L., Chintala N., Adusumilli P.S. (2019). Combination immunotherapy with CAR T cells and checkpoint blockade for the treatment of solid tumors. Cancer Cell.

[34] Michaelides S., Obeck H., Kechur D., Endres S., Kobold S. (2022). Migratory engineering of T cells for cancer therapy. Vaccines.

[35] Andrea A.E., Chiron A., Mallah S., Bessoles S., Sarrabayrouse G., Hacein-Bey-Abina S. (2022). Advances in CAR-T cell genetic engineering strategies to overcome hurdles in solid tumors treatment. Front Immunol.

[36] Marofi F., Achmad H., Bokov D. (2022). Hurdles to breakthrough in CAR T cell therapy of solid tumors. Stem Cell Res Ther.

[37] Hawkins E.R., D’Souza R.R., Klampatsa A. (2021). Armored CAR T-cells: the next chapter in T-cell cancer immunotherapy. Biologics.

[38] Tang L., Pan S., Wei X., Xu X., Wei Q. (2023). Arming CAR-T cells with cytokines and more: innovations in the fourth-generation CAR-T development. Mol Ther.

[39] Yeku O.O., Purdon T.J., Koneru M., Spriggs D., Brentjens R.J. (2017). Armored CAR T cells enhance antitumor efficacy and overcome the tumor microenvironment. Sci Rep.

[40] Patra J.K., Das G., Fraceto L.F. (2018). Nano based drug delivery systems: recent developments and future prospects. J Nanobiotechnology.

[41] Hu B., Ren J., Luo Y. (2017). Augmentation of antitumor immunity by human and mouse CAR T cells secreting IL-18. Cell Rep.

[42] Zannikou M., Duffy J.T., Levine R.N. (2023). IL15 modification enables CAR T cells to act as a dual targeting agent against tumor cells and myeloid-derived suppressor cells in GBM. J Immunother Cancer.

[43] Iwahori K., Kakarla S., Velasquez M.P. (2015). Engager T cells: a new class of antigen-specific T cells that redirect bystander T cells. Mol Ther.

[44] Choi B.D., Yu X., Castano A.P. (2019). CAR-T cells secreting BiTEs circumvent antigen escape without detectable toxicity. Nat Biotechnol.

[45] Gardner T.J., Lee J.P., Bourne C.M. (2022). Engineering CAR-T cells to activate small-molecule drugs in situ. Nat Chem Biol.

[46] Koneru M., Purdon T.J., Spriggs D., Koneru S., Brentjens R.J. (2015). IL-12 secreting tumor-targeted chimeric antigen receptor T cells eradicate ovarian tumors∖textitin vivo. Oncoimmunology.

[47] Frigault M.J., Lee J., Basil M.C. (2015). Identification of chimeric antigen receptors that mediate constitutive or inducible proliferation of T cells. Cancer Immunol Res.

[48] Zimmermann K., Kuehle J., Dragon A.C. (2020). Design and characterization of an “All-in-One” lentiviral vector system combining constitutive anti-GD2 CAR expression and inducible cytokines. Cancers.

[49] Pellegatta S., Savoldo B., Di Ianni N. (2018). Constitutive and TNFα-inducible expression of chondroitin sulfate proteoglycan 4 in glioblastoma and neurospheres: implications for CAR-T cell therapy. Sci Transl Med.

[50] Gardner T.J., Bourne C.M., Dacek M.M. (2020). Targeted cellular micropharmacies: cells engineered for localized drug delivery. Cancers.

[51] Chmielewski M., Abken H. (2015). TRUCKs: the fourth generation of CARs. Expert Opin Biol Ther.

[52] Silveira C.R.F., Corveloni A.C., Caruso S.R. (2022). Cytokines as an important player in the context of CAR-T cell therapy for cancer: their role in tumor immunomodulation, manufacture, and clinical implications. Front Immunol.

[53] Lee E.H.J., Murad J.P., Christian L. (2023). Antigen-dependent IL-12 signaling in CAR T cells promotes regional to systemic disease targeting. Nat Commun.

[54] Hurton L.V., Singh H., Najjar A.M. (2016). Tethered IL-15 augments antitumor activity and promotes a stem-cell memory subset in tumor-specific T cells. Proc Natl Acad Sci U S A.

[55] Trinchieri G., Pflanz S., Kastelein R.A. (2003). The IL-12 family of heterodimeric cytokines: new players in the regulation of T cell responses. Immunity.

[56] Yeku O.O., Brentjens R.J. (2016). Armored CAR T-cells: utilizing cytokines and pro-inflammatory ligands to enhance CAR T-cell anti-tumour efficacy. Biochem Soc Trans.

[57] Liu Y., Di S., Shi B. (2019). Armored inducible expression of IL-12 enhances antitumor activity of glypican-3–targeted chimeric antigen receptor–engineered T cells in hepatocellular carcinoma. J Immunol.

[58] Pegram H.J., Purdon T.J., Van Leeuwen D.G. (2015). IL-12-secreting CD19-targeted cord blood-derived T cells for the immunotherapy of B-cell acute lymphoblastic leukemia. Leukemia.

[59] Zhang L., Morgan R.A., Beane J.D. (2015). Tumor-infiltrating lymphocytes genetically engineered with an inducible gene encoding interleukin-12 for the immunotherapy of metastatic melanoma. Clin Cancer Res.

[60] Zhou W., Miao J., Cheng Z. (2023). Hypoxia-regulated secretion of IL-12 enhances antitumor activity and safety of CD19 CAR-T cells in the treatment of DLBCL. Mol Ther Oncolytics.

[61] Dai S.M., Matsuno H., Nakamura H., Nishioka K., Yudoh K. (2004). Interleukin-18 enhances monocyte tumor necrosis factor α and interleukin-1β production induced by direct contact with T lymphocytes: implications in rheumatoid arthritis. Arthritis Rheum.

[62] Jaspers J.E., Khan J.F., Godfrey W.D. (2023). IL-18–secreting CAR T cells targeting DLL3 are highly effective in small cell lung cancer models. J Clin Invest.

[63] Glienke W., Dragon A.C., Zimmermann K. (2022). GMP-compliant manufacturing of TRUCKs: CAR T cells targeting GD2 and releasing inducible IL-18. Front Immunol.

[64] Perera P.Y., Lichy J.H., Waldmann T.A., Perera L.P. (2012). The role of interleukin-15 in inflammation and immune responses to infection: implications for its therapeutic use. Microbes Infect.

[65] Hoyos V., Savoldo B., Quintarelli C. (2010). Engineering CD19-specific T lymphocytes with interleukin-15 and a suicide gene to enhance their anti-lymphoma/leukemia effects and safety. Leukemia.

[66] Chen Y., Sun C., Landoni E., Metelitsa L., Dotti G., Savoldo B. (2019). Eradication of neuroblastoma by T cells redirected with an optimized GD2-specific chimeric antigen receptor and interleukin-15. Clin Cancer Res.

[67] Zhang Y., Zhuang Q., Wang F. (2022). Co-expression IL-15 receptor alpha with IL-15 reduces toxicity via limiting IL-15 systemic exposure during CAR-T immunotherapy. J Transl Med.

[68] Shi H., Li A., Dai Z. (2023). IL-15 armoring enhances the antitumor efficacy of claudin 18.2-targeting CAR-T cells in syngeneic mouse tumor models. Front Immunol.

[69] Ma X., Shou P., Smith C. (2020). Interleukin-23 engineering improves CAR T cell function in solid tumors. Nat Biotechnol.

[70] Iwakura Y., Ishigame H. (2006). The IL-23/IL-17 axis in inflammation. J Clin Invest.

[71] Duvallet E., Semerano L., Assier E., Falgarone G., Boissier M.C. (2011). Interleukin-23: a key cytokine in inflammatory diseases. Ann Med.

[72] Oppmann B., Lesley R., Blom B. (2000). Novel p19 protein engages IL-12p40 to form a cytokine, IL-23, with biological activities similar as well as distinct from IL-12. Immunity.

[73] Luo H., Su J., Sun R. (2020). Coexpression of IL7 and CCL21 increases efficacy of CAR-T cells in solid tumors without requiring preconditioned lymphodepletion. Clin Cancer Res.

[74] Zhou Z., Li J., Hong J. (2022). Interleukin-15 and chemokine ligand 19 enhance cytotoxic effects of chimeric antigen receptor T cells using zebrafish xenograft model of gastric cancer. Front Immunol.

[75] Nie S., Song Y., Hu K. (2024). CXCL10 and IL15 co-expressing chimeric antigen receptor T cells enhance anti-tumor effects in gastric cancer by increasing cytotoxic effector cell accumulation and survival. Oncoimmunology.

[76] Hughes C.E., Nibbs R.J.B. (2018). A guide to chemokines and their receptors. FEBS J.

[77] Foeng J., Comerford I., McColl S.R. (2022). Harnessing the chemokine system to home CAR-T cells into solid tumors. Cell Rep Med.

[78] Huehls A.M., Coupet T.A., Sentman C.L. (2015). Bispecific T-cell engagers for cancer immunotherapy. Immunol Cell Biol.

[79] Choi B.D., Gerstner E.R., Frigault M.J. (2024). Intraventricular CARv3-TEAM-E T cells in recurrent glioblastoma. N Engl J Med.

[80] Yin Y., Rodriguez J.L., Li N. (2022). Locally secreted BiTEs complement CAR T cells by enhancing killing of antigen heterogeneous solid tumors. Mol Ther.

[81] Cao G., Zhang G., Liu M. (2022). GPC3-targeted CAR-T cells secreting B7H3-targeted BiTE exhibit potent cytotoxicity activity against hepatocellular carcinoma cell in the in vitro assay. Biochem Biophys Rep.

[82] Mun S.S., Meyerberg J., Peraro L. (2023). Dual targeting ovarian cancer by Muc16 CAR T cells secreting a bispecific T cell engager antibody for an intracellular tumor antigen WT1. Cancer Immunol Immunother.

[83] Huang S.W., Pan C.M., Lin Y.C. (2023). BiTE-Secreting CAR-γδT as a dual targeting strategy for the treatment of solid tumors. Adv Sci (Weinheim, Baden-Wurttemberg, Germany).

[84] Branella G.M., Lee J.Y., Okalova J. (2023). Ligand-based targeting of c-kit using engineered γδ T cells as a strategy for treating acute myeloid leukemia. Front Immunol.

[85] Wehrli M., Guinn S., Birocchi F. (2024). Mesothelin CAR T cells secreting anti-FAP/anti-CD3 molecules efficiently target pancreatic adenocarcinoma and its stroma. Clin Cancer Res.

[86] Tomasik J., Jasiński M., Basak G.W. (2022). Next generations of CAR-T cells - new therapeutic opportunities in hematology?. Front Immunol.

[87] Pievani A., Biondi M., Tettamanti S., Biondi A., Dotti G., Serafini M. (2024). CARs are sharpening their weapons. J Immunother Cancer.

[88] Lv Y., Luo X., Xie Z. (2024). Prospects and challenges of CAR-T cell therapy combined with ICIs. Front Oncol.

[89] Zhou J.T., Liu J.H., Song T.T., Ma B., Amidula N., Bai C. (2020). EGLIF-CAR-T cells secreting PD-1 blocking antibodies significantly mediate the elimination of gastric cancer. Cancer Manag Res.

[90] Ping Y., Li F., Nan S. (2020). Augmenting the effectiveness of CAR-T cells by enhanced self-delivery of PD-1-neutralizing scFv. Front Cell Dev Biol.

[91] Suarez E.R., Chang D.K., Sun J. (2016). Chimeric antigen receptor T cells secreting anti-PD-L1 antibodies more effectively regress renal cell carcinoma in a humanized mouse model. Oncotarget.

[92] Wang Y., Fang X., Li M. (2022). Mesothelin CAR-T cells secreting PD-L1 blocking scFv for pancreatic cancer treatment. Cancer Genet.

[93] Yang C., You J., Pan Q. (2023). Targeted delivery of a PD-1-blocking scFv by CD133-specific CAR-T cells using nonviral Sleeping Beauty transposition shows enhanced antitumour efficacy for advanced hepatocellular carcinoma. BMC Med.

[94] Dunn Z.S., Qu Y., MacMullan M., Chen X., Cinay G., Wang P. (2023). Secretion of 4-1BB ligand crosslinked to PD-1 checkpoint inhibitor potentiates chimeric antigen receptor T cell solid tumor efficacy. Hum Gene Ther.

[95] Altvater B., Kailayangiri S., Spurny C. (2023). CAR T cells as micropharmacies against solid cancers: combining effector T-cell mediated cell death with vascular targeting in a one-step engineering process. Cancer Gene Ther.

[96] Lin M.Y., Nam E., Shih R.M. (2024). Self-regulating CAR-T cells modulate cytokine release syndrome in adoptive T-cell therapy. J Exp Med.

[97] Caruana I., Savoldo B., Hoyos V. (2015). Heparanase promotes tumor infiltration and antitumor activity of CAR-redirected T lymphocytes. Nat Med.

[98] Durgin J.S., Thokala R., Johnson L. (2022). Enhancing CAR T function with the engineered secretion of C. perfringens neuraminidase. Mol Ther.

[99] Qu Y., Dunn Z.S., Chen X. (2022). Adenosine deaminase 1 overexpression enhances the antitumor efficacy of chimeric antigen receptor-engineered T cells. Hum Gene Ther.

[100] Zhao Y., Dong Y., Yang S. (2022). Bioorthogonal equipping CAR-T cells with hyaluronidase and checkpoint blocking antibody for enhanced solid tumor immunotherapy. ACS Cent Sci.

